# Impact of fibrinogen levels and modified Glasgow prognostic score on survival of stage III/N2 non-small cell lung cancer patients treated with neoadjuvant therapy and radical resection

**DOI:** 10.1186/s12885-022-10298-9

**Published:** 2022-11-19

**Authors:** Katharina Sinn, Berta Mosleh, Michael Grusch, Walter Klepetko, Konrad Hoetzenecker, Thomas Klikovits, Daniela Gompelmann, Mir Alireza Hoda

**Affiliations:** 1 Department of Thoracic Surgery, Comprehensive Cancer Center, Medical University of Vienna, Waehringer Guertel 18-20, 1090 Vienna, Austria; 2 Center for Cancer Research, Medical University of Vienna, Vienna, Austria; 3 Division of Pulmonology, Department of Medicine II, Medical University of Vienna, Vienna, Austria

**Keywords:** Non-small-cell lung cancer, Fibrinogen, mGPS, N2 disease, Stage III

## Abstract

**Purpose:**

The prognostic value of pretreatment and preoperative fibrinogen plasma levels and the modified Glasgow prognostic score (mGPS) in stage III/N2 non-small cell lung cancer (NSCLC) patients who receive neoadjuvant treatment followed by radical surgery is yet unclear.

**Methods:**

Fibrinogen levels and mGPS of 84 patients with initial stage III/N2 NSCLC, who received neoadjuvant therapy followed by complete surgical resection from 2002 to 2014 were retrospectively analyzed and correlated with clinical parameters and overall survival (OS). Data were analyzed using log-rank and Cox regression analysis adjusted for clinical and pathological factors.

**Results:**

Median serum fibrinogen level after neoadjuvant treatment was 439 mg/dL (IQR 158 mg/dL). Elevated fibrinogen levels (> 400 mg/dL) after neoadjuvant treatment were significantly associated with poorer OS (28.2 months vs. 60.9 months, HR 0.562, *p* = 0.048). Importantly, a decrease in fibrinogen levels after neoadjuvant treatment (*n* = 34) was found to be an independent predictor for favorable OS in multivariate analysis (HR 0.994, *p* = 0.025). Out of 80 patients, 55, 19 and 6 patients had a mGPS of 0, 1 and 2, respectively. Moreover, elevated mGPS after neoadjuvant treatment (mGPS 1–2) showed a non-significant trend for poorer OS compared to mGPS 0 (28.2 vs. 46.5 months, HR 0.587, *p* = 0.066).

**Conclusion:**

Elevated fibrinogen levels after neoadjuvant therapy prior to surgery in stage III/N2 NSCLC patients are associated with significant disadvantage for OS. A decrease in fibrinogen levels after neoadjuvant therapy was found to be a predictor for superior OS in this retrospective patient cohort.

**Supplementary Information:**

The online version contains supplementary material available at 10.1186/s12885-022-10298-9.

## Background

Lung cancer was the leading cause of cancer-related death in both sexes in the world in 2020 [1]. Non-small-cell lung cancer (NSCLC) accounts for approximately 85% of all lung cancers, and 30% of NSCLC patients are diagnosed in locally advanced stage III. Due to the heterogeneity of locally advanced tumors, treatment options differ. The currently available treatment options in patients with stage III N2 disease include neoadjuvant chemotherapy (CHT) or neoadjuvant chemoradiotherapy (CHT/RT) followed by surgery, or definitive CHT/RT ± immunotherapy [2–6]. However, despite all established and novel treatment options, stage III NSCLC patients still have a poor prognosis with a median overall survival (OS) of 42, 22 and 11 months in pathological stages IIIA, IIIB and IIIC, respectively [7]. The response to cancer treatment depends not only on the tumor’s characteristics, but also on the patient’s inflammatory response [8, 9]. Inflammation in the tumor microenvironment contributes to proliferation and tumor survival, angiogenesis, invasion, metastasis, subversion of adaptive immunity, reduced response to hormones and therapeutic agents [10]. Cancer-related systemic symptoms such as weight loss (i.e. cachexia and anorexia) and anemia are mostly inflammatory-driven and thus either part of the host’s innate response against cancer or result from a direct cytokine release from the tumor itself [11]. Accordingly, it has been reported that tumor-associated systemic inflammation and malnutrition have a prognostic relevance in many solid tumors [12].

Fibrinogen is an acute phase protein, produced by the liver in response to pro-inflammatory cytokines, characterized by its elevation in patients with malignant and inflammatory diseases [13]. Further, fibrinogen has a central role in the coagulation cascade and during wound healing [14]. Several studies have reported that an increased fibrinogen level is a well-established independent negative prognostic factor in different solid tumors [15–19]. In addition, a selective combination of elevation of serum CRP and reduction of serum albumin, known as the modified Glasgow prognostic score (mGPS) has been reported to have prognostic value in several malignancies [20, 21].

Therefore, the aim of this study was to examine the clinical prognostic value of fibrinogen levels and the mGPS in patients with initial stage III/N2 NSCLC who received neoadjuvant therapy followed by radical resection. The association between neoadjuvant treatment-induced changes in fibrinogen levels and mGPS and disease free survival (DFS) and OS were also analyzed.

## Methods

### Study design

In this single-center, retrospective cohort study, consecutive patients with clinical stage III/N2 NSCLC undergoing curative-intent surgery after neoadjuvant therapy at the Department of Thoracic Surgery at the Medical University of Vienna between 2002 and 2014 were included (Supp. Fig. 1). Date of diagnosis was taken as the time of the first radiological examination that raised a suspicion of malignancy. All cases were (re)staged by using the 8th edition of the TNM lung cancer classification [22]. Laboratory reports from all patients were reviewed for CRP, albumin and fibrinogen at time of diagnosis and admission for surgery. Patients suffering from autoimmune disease or post-stenotic pneumonia were excluded from this study. Patients with comorbidities were identified and objectively assessed using the well established Charlson Comorbidity index (CCI) [23–26]. The study was approved by the ethics committee of the Medical University of Vienna according to the declaration of Helsinki (EK No 1448/2017). Outcomes have been reported according to the “Strengthening the reporting of cohort studies in surgery (STROCSS)” 2019 Guidelines [27].

### Modified Glasgow prognostic score

The cutoff values for CRP (1 mg/dL) and serum albumin (35 g/L) were based on the paper by Proctor et al. [28]. Serum CRP levels were measured by the modified latex-enhanced immunoturbidimetric assay using CRP Latex kit (Olympus Life and Material Science Europe). The values for the mGPS were defined as follows: mGPS 0, CRP ≤ 1 mg/dL and any albumin; mGPS 1, CRP > 1 mg/dL and albumin ≥ 35 g/L; mGPS 2, CRP > 1 mg/dL and albumin < 35.

### Evaluation, treatment and follow-up

All patients in this study were histologically verified and had clinically or biopsy-proven N2 disease and were, therefore treated with neoadjuvant therapy. The decision which patients with clinical stage III/N2 NSCLC were able to undergo surgery after neoadjuvant treatment including induction CHT/RT or CHT in case of response to neoadjuvant therapy was taken by a multidisciplinary tumor board with the participation of a thoracic surgeon, pulmonologist, radiation oncologist, oncologist and radiologist. N2 disease was partially verified by endobronchial ultrasound-guided transbronchial needle aspiration (EBUS-TBNA) or mediastinoscopy. However, in patients without invasive mediastinal staging, cN2 LN involvement was defined if N2 LNs were FDG-PET positive or significantly enlarged (> 15 mm in diameter at CT scan) and documented after consultation in a tumor board. After neoadjuvant treatment including CHT/RT or CHT alone, the CT scan performed for re-staging was discussed again by a multidisciplinary team and the decision for surgery was made. Histology, tumor grade and LN involvement were assessed at the Department of Pathology, Medical University of Vienna. Depending on the final histology report and pathological stage, patients received further adjuvant therapy. Follow-up visits included regular CT scans to detect disease recurrence or secondary primary tumors. The time point of recurrence was defined as the date of imaging. DFS was calculated from time of surgery to the time of recurrence. OS was calculated as the time from surgery to the date of death from any cause. Living patients were censored at the time of last contact.

### Data collection and statistical analysis

Data were retrieved from the institutional thoracic surgery database as well as from the patient’s documentation system of the Medical University of Vienna. Additionally, data were collected from the referring hospital documentation system and dates of death were reconciled with the death records of “Statistik Austria”.

Statistical analyses were performed using the SPSS software (IBM SPSS, IBM Corp., Armonk, NY, USA). Normally distributed data were presented as mean ± standard deviation (SD) and non-normal distributions as median (range). Two dependent groups with normal distribution were compared by the paired Student’s t-test. The Chi-Square test was used for testing differences between two categorical variables. OS and DFS were analyzed using the Kaplan–Meier method and compared with the log-rank test. Univariate and multivariate Cox’s regression was used to calculate hazard ratios (HRs) for DFS and OS and to explore prognostic clinical factors. Following variables have been selected for multivariate analysis: age (continuously), gender, CCI (0–1 vs > 2), clinical stage (IIIA vs IIIB), histological subtype (adenocarcinoma vs others), type of induction therapy (CHT/RT vs CHT), fibrinogen, mGPS. N2 downstaging. Statistical significance was defined as a two-tailed *p*-value of < 0.05. Relative risks are reported with 95% confidence intervals.

All graphical illustrations were created using GraphPad Prism (GraphPad Prism, GraphPad Software, La Jolla, CA, USA).

## Results

In total, 84 patients with clinical stage III/N2 NSCLC, who received neoadjuvant treatment were included (CHT/RT *n* = 34, 40%; CHT *n* = 50, 60%) (Table 1, Supp. Fig. 1). The majority of patients were men (*n* = 57, 68%; female *n* = 27, 32%), with a mean age of 60.8 years (± 9.0 years) and a mean BMI of 26.0 kg/m^2^ (± 4.8) at time of surgery. Sixty-eight patients (81%) were initially diagnosed with stage IIIA, N2, whereas 16 patients (19%) were diagnosed with stage IIIB, N2 (Table 1). The most frequent histology was adenocarcinoma in 41 patients (49%), followed by 37 patients (44%) with squamous cell carcinoma (Table 1). In 13 patients (15.5%) a history of previous malignancies is known (Table 2). Comorbidities and lung function parameters of all patients are listed in Table 2.

**Table 1:** Clinicopathologic features in patients with clinical stage III/N2 NSCLC

All patients (*n* = 84)	
**Age** (median $\pm$ SD)	61.5 $\pm$ 8.98
**Gender** (Male/ Female)	57/27
**Comorbidities**	59 (70.2%)
**FEV1** < 60% (*n* = 79^a^)	9 (10.7%)
**Initial staging**	
- IIIA	68 (81.0%)
- IIIB	16 (19.0%)
**Neoadjuvant Treatment**	
- CHT	50 (60%)
- CHT/RT	34 (40%)
**Type of resection**	
- Pneumonectomy	27 (32.2%)
- Bilobectomy	6 (7.1%)
- Lobectomy	50 (59.5%)
- Segmental resection	1 (1.2%)
**Extended resection**^b^	37 (44.1%)
**Histological subtype**	
- Adenocarcinoma	41 (48.8%)
- Squamous cell carcinoma	37 (44.0%)
- Large cell carcinoma	6 (7.2%)
**Pathological staging**	
- Complete response	9 (10.7%)
- I	26 (31.0%)
- II	24 (28.6%)
- III	25 (29.8%)
- IV	0 (0%)
**N2 Downstaging** (pN0-1)	63 (75.0%)
**Postop. Complications (all)**	16 (19.0%)
- Rec. nerve palsy	6 (7.1%)
- Chylothorax	6 (7.1%)
- Wound infection	2 (2.4%)
- Bleeding	1 (1.2%)
- Bronchopleural fistula	0 (0%)
- Other	3 (3.6%)
**Adjuvant Treatment**	26 (31.0%)
- aCHT	17 (20.2%)
- aRT	4 (4.8%)
- aCHT/RT	5 (6.0%)

*CHT/RT* Chemoradiotherapy, *CHT* Chemotherapy, *aCHT* adjuvant chemotherapy, *aRT* adjuvant radiotherapy, *aCHT/RT* adjuvant chemoradiotherapy, Data shown in parentheses are column percentages

^a^In 5 cases, FEV1 (Forced Expiratory Volume in the first second) data were not available

^b^Extended resections include bronchial sleeve, vascular sleeve or resections of the chest wall, diaphragm or pericardium and were stated additionally to type of resection

**Table 2:** Comorbidities and lung function parameters prior to surgery in patients with clinical stage III/N2 NSCLC

All patients (*n* = 84)	
Comorbidities (Yes, n, %)	59 (%)
COPD ^a^ (n, %)	34 (38.2%)
Diabetes mellitus (n, %)	12 (11.7%)
Arterial hypertension (n, %)	35 (26.5%)
Cardiovascular disease (n, %)	17 (14.7%)
Previous Malignancies (n, %)	13 (8.8%)
FEV1%^a^ (Mean ± SD)	79.6% ± 20.8
FVC% ^a^ (Mean ± SD)	86.3% ± 15.7
pO2 ^a^ (mmHg, Mean ± SD)	72.3 ± 16.4
pCO2 ^a^ (mmHg, Mean ± SD)	40.4 ± 12.0

*COPD* Chronic obstructive pulmonary disease, *FEV1* Forced expiratory volume in the first second, *FVC* Forced vital capacity, Data shown in parentheses are column percentages

^a^In 5 cases data were not available

Lobectomy was the most frequently performed resection type (*n* = 50, 60%), followed by pneumonectomy (*n* = 27, 32%), bilobectomy (*n* = 6, 7%), and sublobar resection (*n* = 1, 1%). Nodal downstaging (from N2 disease at diagnosis to pN0/1 confirmed by pathological result from surgical resection) was achieved in 75%, whereby 44.5% (*n* = 28) and 55.6% (*n* = 35) of these patients received CHT/RT or CHT alone, respectively (*p* = 0.199) (Table 1). Twenty-one of our 84 (25%) patients had persistent positive mediastinal LNs, whereas 66.7% (*n* = 14) of these patients had single station N2 disease.

Pathological complete responses (ypT0, ypN0) were observed exclusively in the CHT/RT cohort: 26% (*n* = 9) vs. none in the CHT alone group, *p* =  < 0.001. Eighteen percent (*n* = 6) and 40% (*n* = 20) of patients in the CHT/RT and CHT groups received adjuvant therapy, respectively (*p* = 0.021, Table 1).

### Fibrinogen level and mGPS at time of surgery

After neoadjuvant therapy, at time of admission for surgery, the median fibrinogen level was 439.0 mg/dL (IQR 158, *n* = 84). The majority of patients (*n* = 51, 60.7%) had clinically relevant elevated plasma fibrinogen levels (≥ 400 mg/dL), whereas only 26 patients (31%) had an elevated CRP > 1 mg/dL. Additionally, only 7 patients (8.8%) had albumin levels < 35 g/L before surgery (data of 4 patients missing). There was no significant difference in the investigated laboratory parameters between patients who received combined chemotherapy/radiotherapy or chemotherapy alone.

Nine patients showed complete pathological response (pCR) at surgery, thereof 6/9 patients had fibrinogen levels below the cut-off level of 400 mg/dL at time of surgery (*p* = 0.075). In contrast, 21 patients had persistent N2 disease, 15/21 patients (71.4%) had high fibrinogen levels (≥ 400 mg/dL, *p* = 0.299).

After neoadjuvant treatment 55 patients (68.8%) had a mGPS of 0, 19 patients (23.8%) had a mGPS of 1 and 6 patients (7.5%) had a mGPS of 2 (in 4 patients albumin levels were missing). All 9 patients with pCR had a mGPS of 0 at time of surgery (*p* = 0.100). The mGPS values of patients with persistent N2 disease at the time of admission for surgery were as follows (data in one patient missing): 0: 14/21 (66.7%); 1: 5/21 (23.8%); 2: 1/21 (4.8%), (*p* = 0.879).

### Association between fibrinogen level and mGPS assessed prior to surgery and survival

Median follow-up time for all patients was 33 months (IQR 46.0), for surviving patients it was 58 months (IQR 34.9). Seven patients died intercurrently after a median time after surgery of 26.7 months (IQR 35.9) due to myocardial infarction (*n* = 2), suicide (*n* = 1), sepsis (*n* = 1), and tonsillar carcinoma (*n* = 1). The cause of death remained unknown for two patients.

**Fig. 1:**
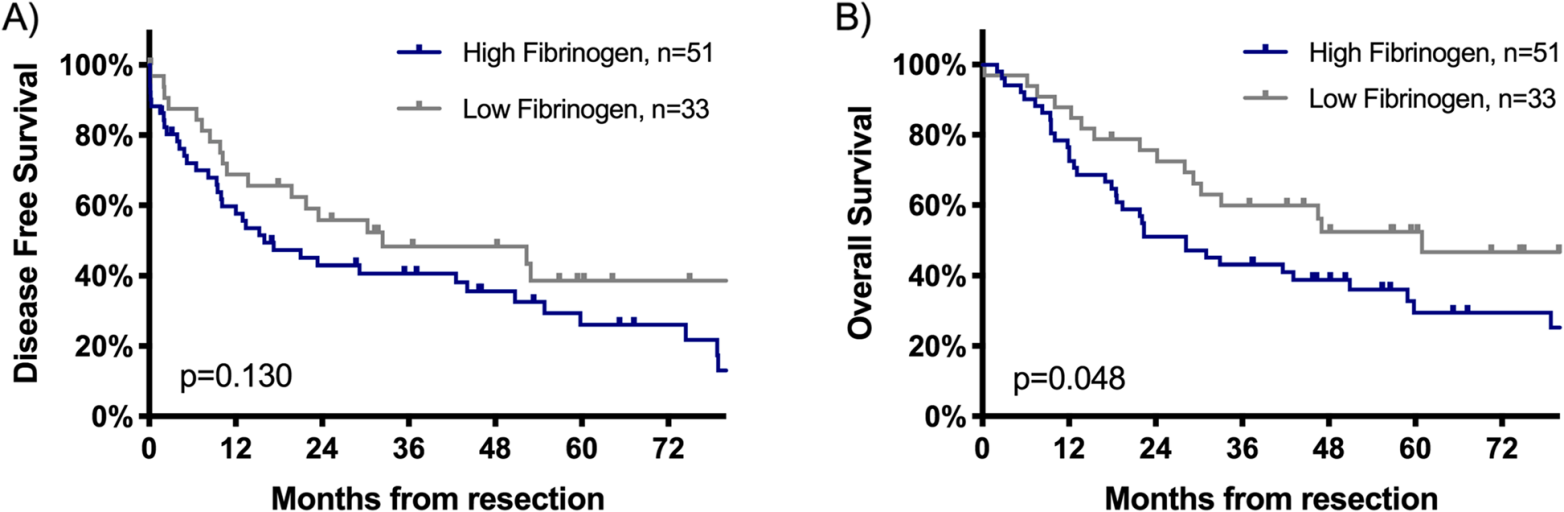
Comparison of survival outcomes for the DFS (**A**) and OS (**B**) in surgically treated stage III/N2 NSCLC patients after neoadjuvant therapy according to fibrinogen levels (Median DFS and OS were 15.97 vs 32.37 months and 28.17 vs 60.9 months, respectively; HR 0.653, 95% CI 0.374–1.139, *p* = 0.130; and HR 0.562, 95% CI 0.315–1.004, *p* = 0.048). Fibrinogen cut off value was 400 mg/dL

**Fig. 2:**
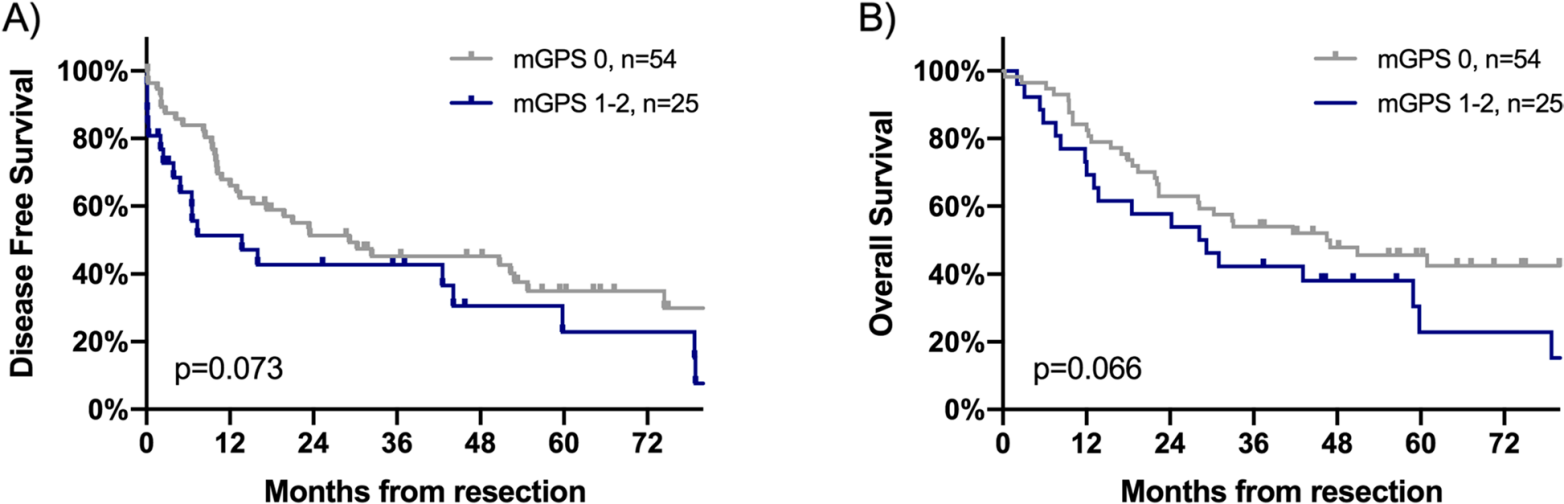
Comparison of survival outcomes for the DFS (**A**) and OS (**B**) in surgically treated stage III/N2 NSCLC patients after neoadjuvant therapy according to mGPS (Median DFS and OS were 7.3 vs. 29.17 months and 28.17 vs. 46.47 months, respectively; HR 0.595, 95% CI 0.335–1.056, *p* = 0.073; HR 0.587, 95% CI 0.331–1.042, *p* = 0.066). The values for the mGPS were defined as follows: mGPS 0, CRP ≤ 1 mg/dL and any albumin; mGPS 1, CRP > 1 mg/dL and albumin ≥ 35 g/L; mGPS 2, CRP > 1 mg/dL and albumin < 35

After neoadjuvant treatment at time of admission for surgery, patients with fibrinogen levels > 400 mg/dL had a significantly decreased median OS of 28.2 months as compared to those with low fibrinogen level (60.9 months, HR 0.562, 95% CI 0.315–1.004, *p* = 0.048, Fig. 1). Nevertheless, there was no significant difference in the DFS of patients with high versus low plasma fibrinogen levels (cut off value 400 mg/dL, 16.0 vs 32.4 months, respectively, HR 0.653, 95% CI 0.374–1.139, *p* = 0.130, Fig. 1). Furthermore, for patients with elevated mGPS after neoadjuvant treatment at time of surgery (mGPS 1–2) there was a trend for shorter median OS compared to patients with normal mGPS (mGPS 0), but without statistical significance (28.2 vs. 46.5 months, HR 0.587, 95% CI 0.331–1.042, *p* = 0.066, Fig. 2). There was also no statistically significant difference between the median DFS of patients with elevated mGPS and normal mGPS (7.3 vs. 29.2 months HR 0.595, 95% CI 0.335–1.056, *p* = 0.073, Fig. 2).

In a multivariate analysis, fibrinogen, age (continuously), gender, CCI (0–1 vs ≥ 2), clinical stage (IIIA vs IIIB), histological subtype (adenocarcinoma vs others) and type of induction therapy (CHT/RT vs CHT) were evaluated as predictors for OS. Only type of induction therapy was found to be an independent prognostic factor after multivariate analysis (HR 0.444, 95% CI 0.234–0.843, *p* = 0.013), whereas all other parameters including fibrinogen levels at time of surgery had no independent prognostic impact on OS in our study population.

### Changes of fibrinogen level and mGPS prior to and following neoadjuvant treatment

At time of diagnosis fibrinogen levels were available in 34 patients (40.5%). Median fibrinogen level was 508.5 mg/dL (IQR 222), whereby most patients (*n* = 23/34, 67.6%) had clinically relevant elevated plasma fibrinogen levels (≥ 400 mg/dL). In 59.5% of patients the CRP was elevated > 1 mg/dL (*n* = 44/74 data available), whereas only in 5/45 patients (11.1%) album levels < 35 g/L were present.

At time of diagnosis, mGPS was assessed in 45 patients (53.7%). Out of the 45 patients, 17 (37.8%) had a mGPS of 0, 23 (51.1%) had a mGPS of 1 and 5 (11.1%) had a mGPS of 2.

Comparison of fibrinogen levels prior to and following neoadjuvant treatment was assessed in 34 patients. A decrease of fibrinogen level and a reduction of mGPS were observed in 64.7% (22/34) and 31.1% (14/45) of patients, respectively. Overall, neoadjuvant treatment led to a significant change in fibrinogen level (*p* < 0.001) and mGPS (*p* < 0.001).

### Association between change of fibrinogen levels and mGPS prior to and following neoadjuvant treatment and survival

Multivariate analysis (difference in fibrinogen levels, change of mGPS, N2 downstaging, type of induction therapy [CHT/RT vs CHT], cStage, age [continuously], histological subtype [adenocarcinoma vs others]), revealed that changes in fibrinogen levels and type of induction therapy were independent predictors of OS (*p* = 0.025, HR 0.994, 95% CI 0.989–0.999 and *p* = 0.019, HR 0.099, 95% CI 0.014–0.679, Table 3).

**Table 3:** Cox-Regression analysis for disease free survival and overall survival in patients with clinical stage III/N2 NSCLC

Cox-Regression	Disease free survival			Overall survival		
	**HR**	**95% CI**	***p*-value**	**HR**	**95% CI**	***p*-value**
Difference in fibrinogen^a^	0.996	0.991–1.001	0.136	0.994	0.989–0.999	0.025
Decrease in mGPS^b^	0.501	0.125–2.004	0.328	0.414	0.088–1.949	0.265
N2 downstaging	0.796	0.297–2.130	0.649	0.563	0.194–1.636	0.291
CHT/RT vs CHT	0.170	0.033–0.876	0.034	0.099	0.014–0.679	0.019
cStage	0.436	0.102–1.857	0.262	0.372	0.077–1.800	0.219
Histology (ADC vs other)	0.916	0.306–2.737	0.875	0.621	0.200–1.929	0.410
Age (continuously)	0.998	0.940–1.061	0.955	1.052	0.971–1.141	0.215

*mGPS* Modified glasgow prognostic score, *CHT/RT* Chemoradiotherapy, *CHT* Chemotherapy, *ADC* Adenocarcinoma

^a,b^Data were available for fibrinogen in 34 patients (40.5%) and mGPS in 45 patients (53.7%) at time of diagnosis and prior surgery

## Discussion

This retrospective study demonstrates that patients with stage III/N2 NSCLC and elevated fibrinogen levels after neoadjuvant therapy prior to surgery are associated with significant shorter OS. Further, a decrease in fibrinogen levels after neoadjuvant therapy was found to be a predictor for superior OS.

The relationship between inflammation and cancer is well investigated [8, 29]. Cancer-related systemic symptoms such as weight loss (i.e. cachexia and anorexia) and anemia are mostly inflammatory-driven and thus either part of the host’s innate response against cancer or result from a direct cytokine release from the tumor itself [11]. Accordingly, it has been reported that tumor-associated systemic inflammation has a prognostic relevance in many solid tumors [12]. Fibrinogen is an acute phase protein with elevated plasma levels found in patients with malignant and inflammatory diseases. Increased fibrinogen level is a well-established independent negative prognostic factor in different solid tumors [15–19]. A selective combination of CRP and serum albumin (termed the mGPS) has also been reported to have prognostic value in several malignancies [20, 21].

Various trials evaluated the role of preoperative fibrinogen in patients with NSCLC undergoing surgical resection [30, 31] and reported conflicting findings on the impact of fibrinogen level and lung cancer prognosis. Different studies confirmed that plasma fibrinogen was associated with tumor histology and pathological T stage and fibrinogen was shown to be an independent prognostic biomarker in operable NSCLC [30, 31]. In contrast to these findings, Ohara et al. found, that high plasma levels were predictors of a poor prognosis in patients with surgically resected NSCLC according to a univariate analysis, but not in multivariate analysis [32]. All these trials focused on treatment-naïve patients with lung cancer at stage I-III undergoing surgical resection.

In contrast to these previous studies, our trial assessed the role of fibrinogen in patients with lung cancer stage III treated initially by neoadjuvant therapy prior to surgical resection. In this selected patient cohort, high fibrinogen plasma levels after neoadjuvant therapy but prior to surgery were significantly associated with shorter OS (but not DFS) in univariate analysis, but were not predictive for a poor prognosis of lung cancer patients in a multivariate model. Thus, these findings were similar to those of Ohara et al. although the study cohorts differed in terms of cancer stage and neoadjuvant treatment [32].

To our knowledge, there is no study so far which analyzed the changes in fibrinogen levels and mGPS before and after neoadjuvant treatment in resectable NSCLC. Moreover, our study investigated the relationship between the fibrinogen level and mGPS prior to and following neoadjuvant treatment and survival after radical resection. Changes in fibrinogen level were found to be an independent predictor for superior OS. Therefore, these findings should encourage the initiation of further large, randomized trials to confirm the hypothesis that persistent elevated fibrinogen levels represent a poor prognosis for patients with advanced NSCLC and multimodality treatment.

Besides fibrinogen level, we evaluated the mGPS in patients with stage III lung cancer. There was a trend for shorter median OS in patients with elevated GPS compared to patients with normal mGPS, but without statistical significance. Similar results were found in one meta-analysis [33]. Even though the authors observed a significant association between elevated mGPS and poorer OS in lung cancer patients receiving chemotherapy or other palliative treatment, the association between mGPS and poorer OS was non-significant in patients undergoing surgery.

Our study has several limitations. First, it is a retrospective study, which was mainly performed at the Department for Thoracic Surgery and selected patients were referred after neoadjuvant treatment and after discussion in an interdisciplinary tumor board from different centers. We do not know how many patients with clinical stage III/N2 disease started induction therapy but were not referred for surgery due to disease progression or deterioration of general condition. However, all patients who actually underwent surgery were included. Also, in some patients the diagnosis of N2 positive LNs had not been histologically confirmed at time of diagnosis. Accordingly, enlarged LNs (> 15 mm short axis) found in the chest CT-scan with contrast medium, or increased FDG uptake at PET-CT were classified as cN2 disease and treatment was indicated within a multidisciplinary tumor board.

Another limitation of this work is the small number of patients, especially the even smaller number with a complete data set (all parameters at time of diagnosis and after neoadjuvant therapy prior to surgery). Despite the rather small patient number, we could identify that the decrease of fibrinogen levels after neoadjuvant treatment is an independent prognostic factor.

## Conclusions

Summarizing, while increased fibrinogen level prior to surgery is a negative predictor for OS, a decrease in fibrinogen level after neoadjuvant treatment at time of surgery predicts for better OS of stage III N2 lung cancer patients. However, prospective studies are necessary to confirm these encouraging findings.

## Supplementary Information

**Additional file 1:** **Suppl. Fig. 1.** Consort diagram to demonstrate the selection of stage III/N2 NSCLC patients for surgery after neoadjuvant treatment in this study. Where patients were excluded, the reasons for exclusion are indicated. cN1, clinical N1 disease; cN3, clinical N3 disease.

## Data Availability

The datasets generated and analyzed during the current study are available from the corresponding author on reasonable request. The data are not publicly available due to privacy and ethical restrictions.

## Abbreviations

CCI: Charlson comorbidity index
CHT/RT: Chemoradiotherapy
CHT: Chemotherapy
CI: Confidence interval
CT: Computerized tomography
DFS: Disease-free survival
FDG-PET: Fluorodeoxyglucose-positron emission tomography
HR: Hazard ratio
IQR: Interquartile range
LN: Lymph node
mGPS: Modified Glasgow prognostic score
NSCLC: Non-small cell lung cancer
OS: Overall survival
pCR: Pathological complete response
SD: Standard deviation
