# Supplementary material for: Impact of fibrinogen levels and modified Glasgow prognostic score on survival of stage III/N2 non-small cell lung cancer patients treated with neoadjuvant therapy and radical resection
Source: BMC Cancer. 2022 Nov 19;22:1197. doi: 10.1186/s12885-022-10298-9 (PMC9675967; doi:10.1186/s12885-022-10298-9)
Supplement: Supplementary file 1 — Additional file 1: Suppl. Fig. 1. Consort diagram to demonstrate the selection of stage III/N2 NSCLC patients for surgery after neoadjuvant treatment in this study. Where patients were excluded, the reasons for exclusion are indicated. cN1, clinical N1 disease; cN3, clinical N3 disease. [file 12885_2022_10298_MOESM1_ESM.pdf]

Suppl. Fig. 1

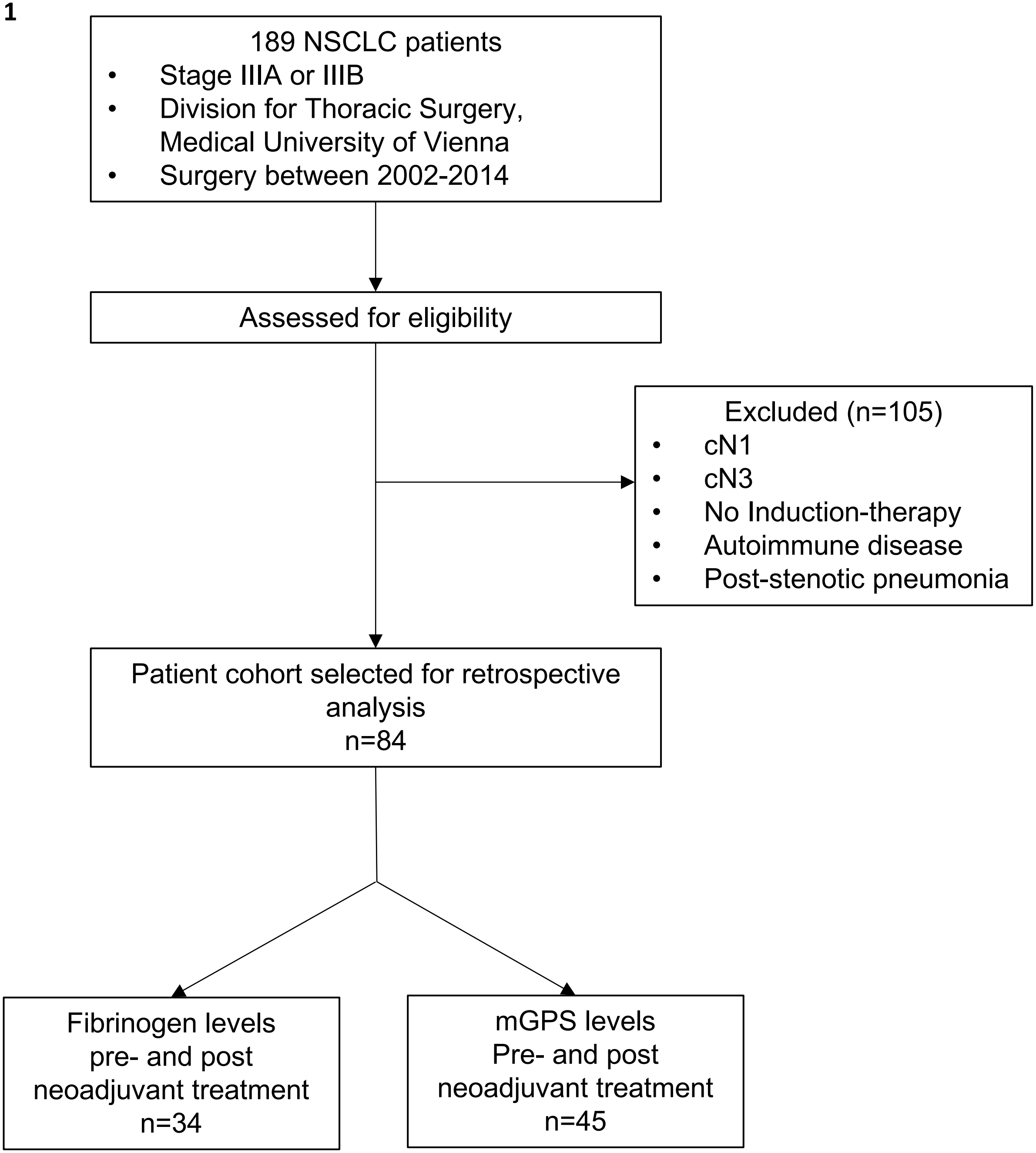

Consort diagram to demonstrate the selection of stage III/N2 NSCLC patients for surgery after neoadjuvant treatment in this study. Where patients were excluded, the reasons for exclusion are indicated. cN1, clinical N1 disease; cN3, clinical N3 disease
